# Supplementary figures and images for: Beyond core object recognition: Recurrent processes account for object recognition under occlusion
Source: PLoS Comput Biol. 2019 May 15;15(5):e1007001. doi: 10.1371/journal.pcbi.1007001 (PMC6538196; doi:10.1371/journal.pcbi.1007001)

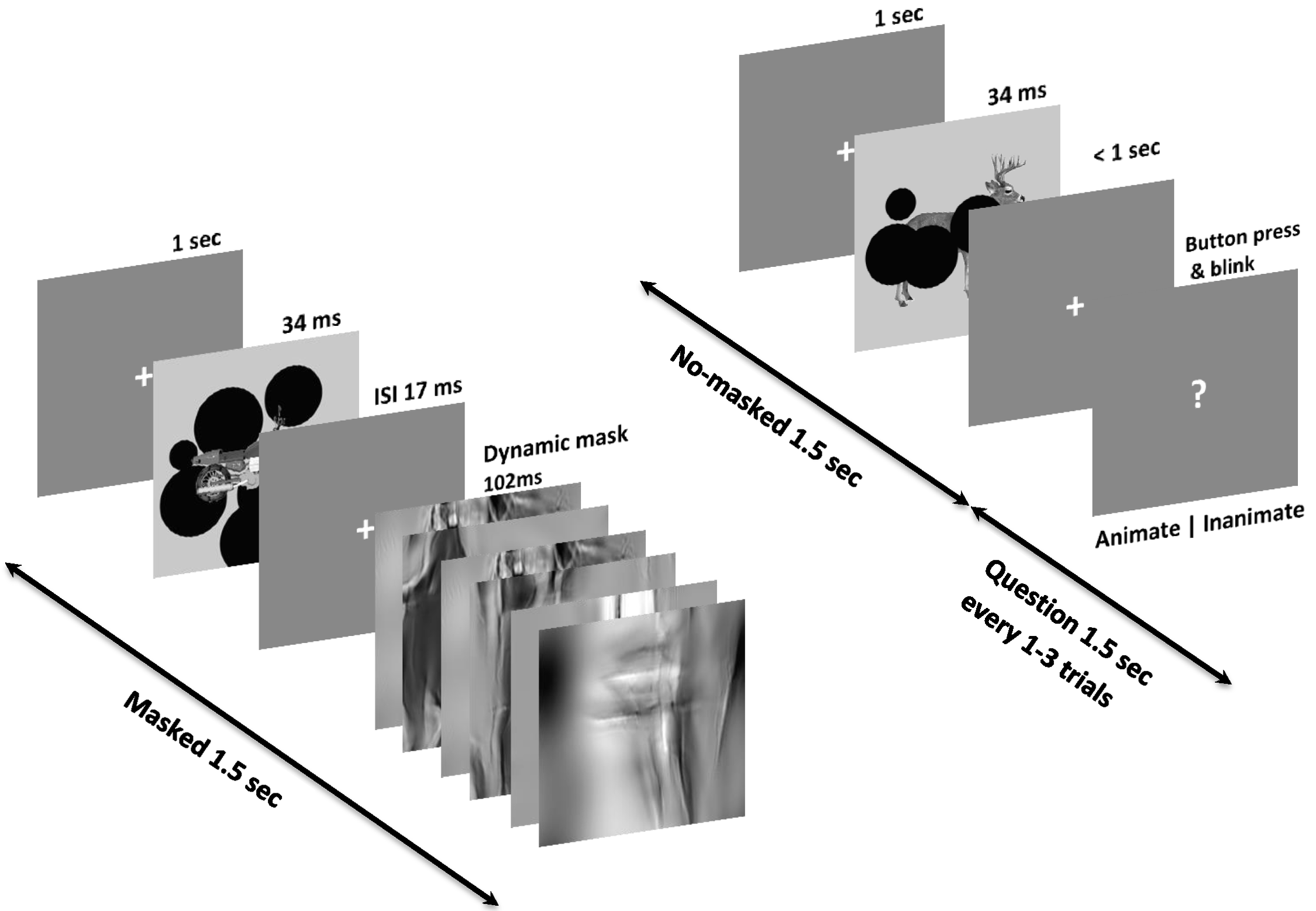

Supplement: S1 Fig — The experiment was divided into two types of trials: mask and no mask trials, shown in random order. Each trial started by a fixation of 1 sec followed by a target stimulus presented for 34ms. In the mask trials, after a short inter-stimulus-interval (ISI) of 17ms, a dynamic mask of 102ms duration was presented. Every 1–3 trials (average = 2) a question mark appeared on the screen. Subjects were asked to select whether the last image was animate or inanimate. They were also instructed to restrict their blinking (and swallowing) to the question-mark trials. (TIF) [file pcbi.1007001.s001.tif]

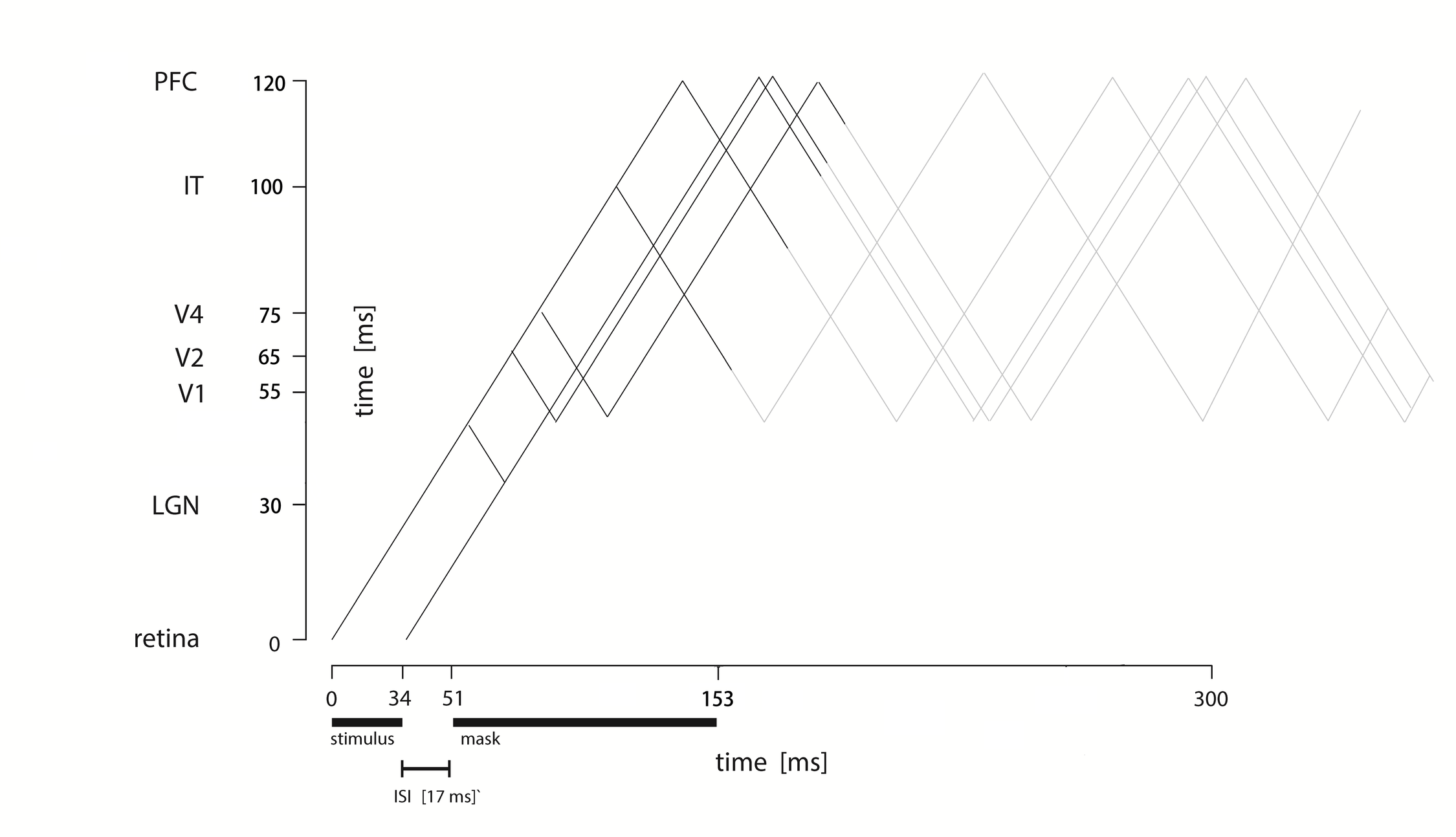

Supplement: S2 Fig — Earliest responses reaching each of the visual areas from V1 to IT are indicated by the oblique lines, when a stimulus is on for 34ms, followed by an ISI of 17 ms, followed by a mask. The grey shaded area indicates the effect of mask when it disrupts the information that is being fed back from higher visual areas to lower visual areas. The approximate timings are set according to human [38, 50, 105] and non-human (i.e. macaque) studies [4] controlling for the fact that the macaque cortex is smaller, with a shorter neural distance and therefore faster transmission of visual information [37]. (TIF) [file pcbi.1007001.s002.tif]

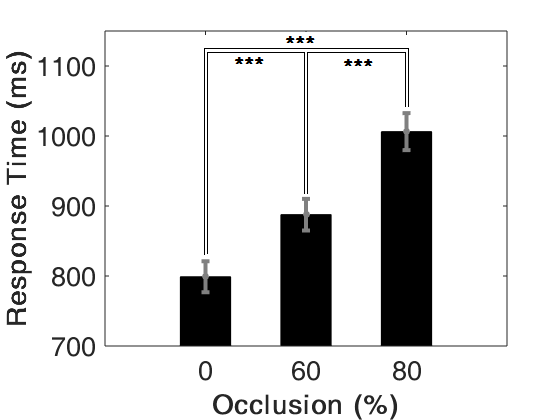

Supplement: S3 Fig — The results are averaged over n = 15 human participants. Error bars represent SEM. Significant difference between occlusion levels are indicated by stars (signrank test). *** = p<0.001. (TIF) [file pcbi.1007001.s003.tif]

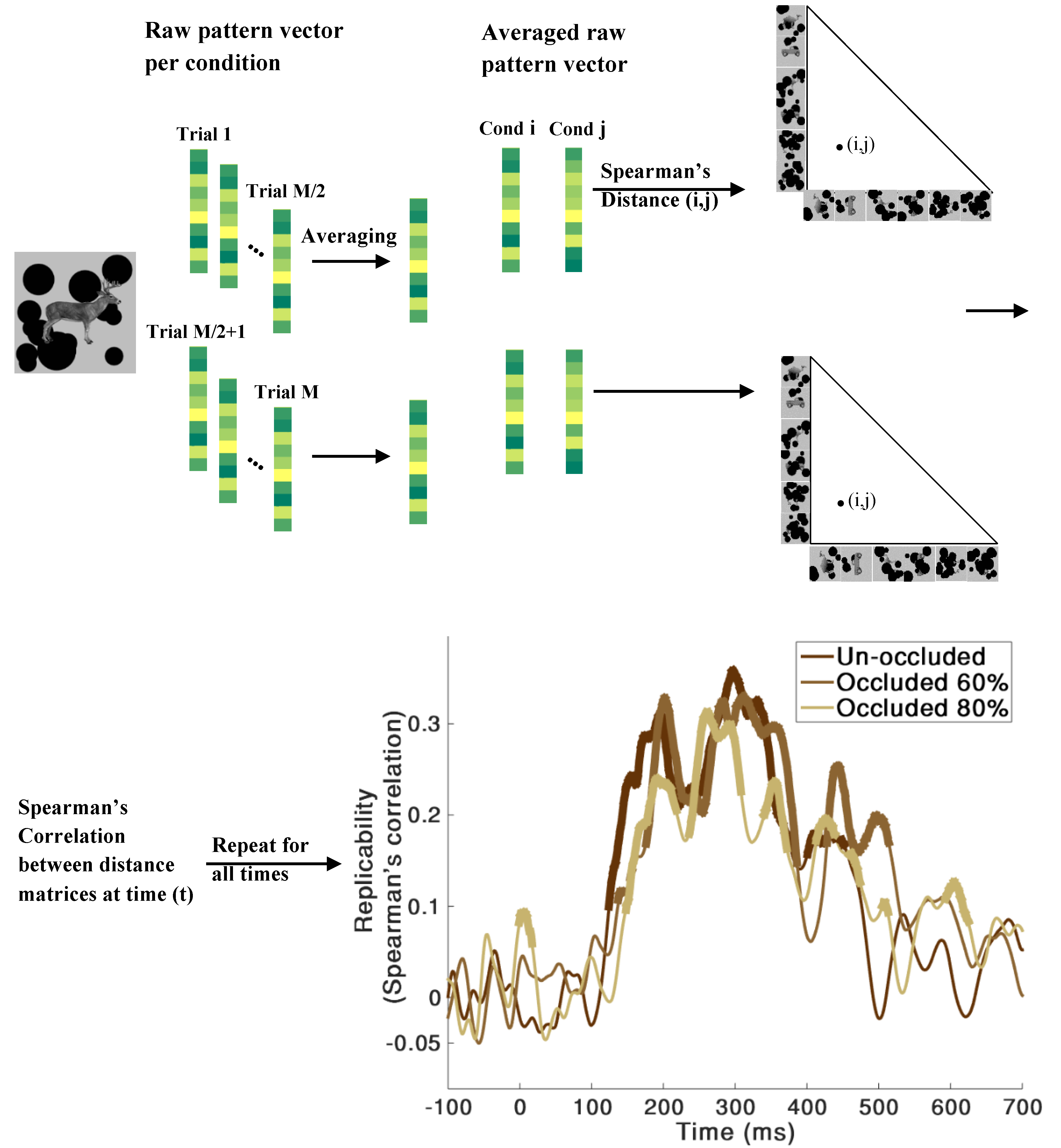

Supplement: S4 Fig — The MEG trials for each condition (i.e. 0% occlusion, 60% occlusion, and 80% occlusion) were divided into two halves, the replicability is measured as the correlation between these two halves. Thicker lines indicate significantly above chance correlations (right sided sign-rank test, FDR corrected across time, p<0.05). No significant difference was observed between the replicability of different conditions (two-sided signrank test, FDR-corrected across time), thus indicating that different conditions do not differ in their level of noise. In more details, for each condition, we randomly split M = 64 trial repetitions into two groups of 32 trials. Distance matrices were then calculated for average raw pattern vectors of each group by computing pairwise dissimilarity (1-correlation) between the patterns (12x12 matrices; 12 experimental stimuli). Spearman’s R was used as the replicability measure between these two split-half matrices across time. (TIF) [file pcbi.1007001.s004.tif]

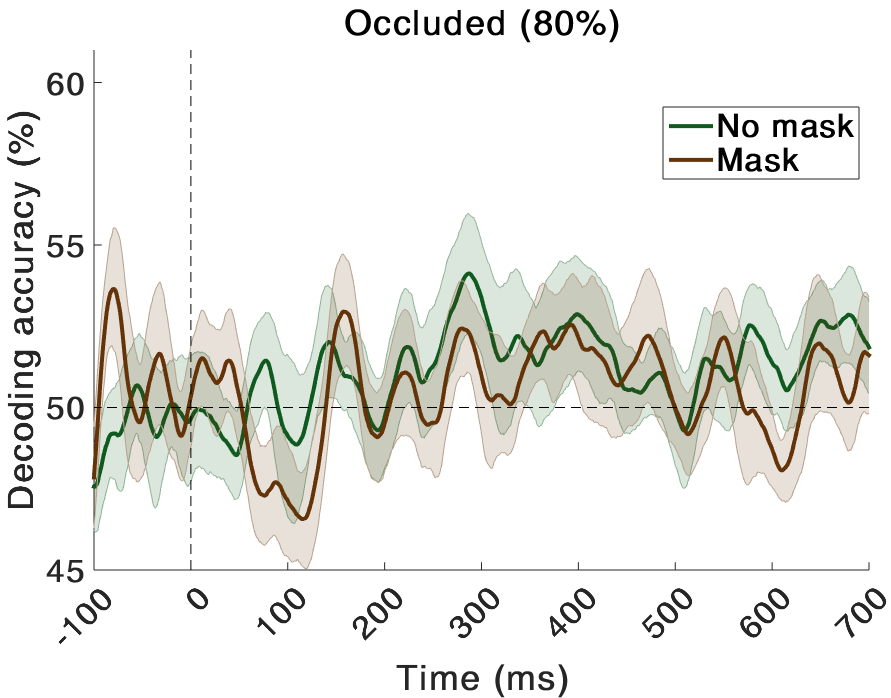

Supplement: S5 Fig — Shaded error bars represent standard error of the mean (SEM). Decoding accuracy was not significantly above chance at any time-point for both mask and no mask (right-sided signrank test, FDR-corrected across time, p < 0.5). (TIF) [file pcbi.1007001.s005.tif]

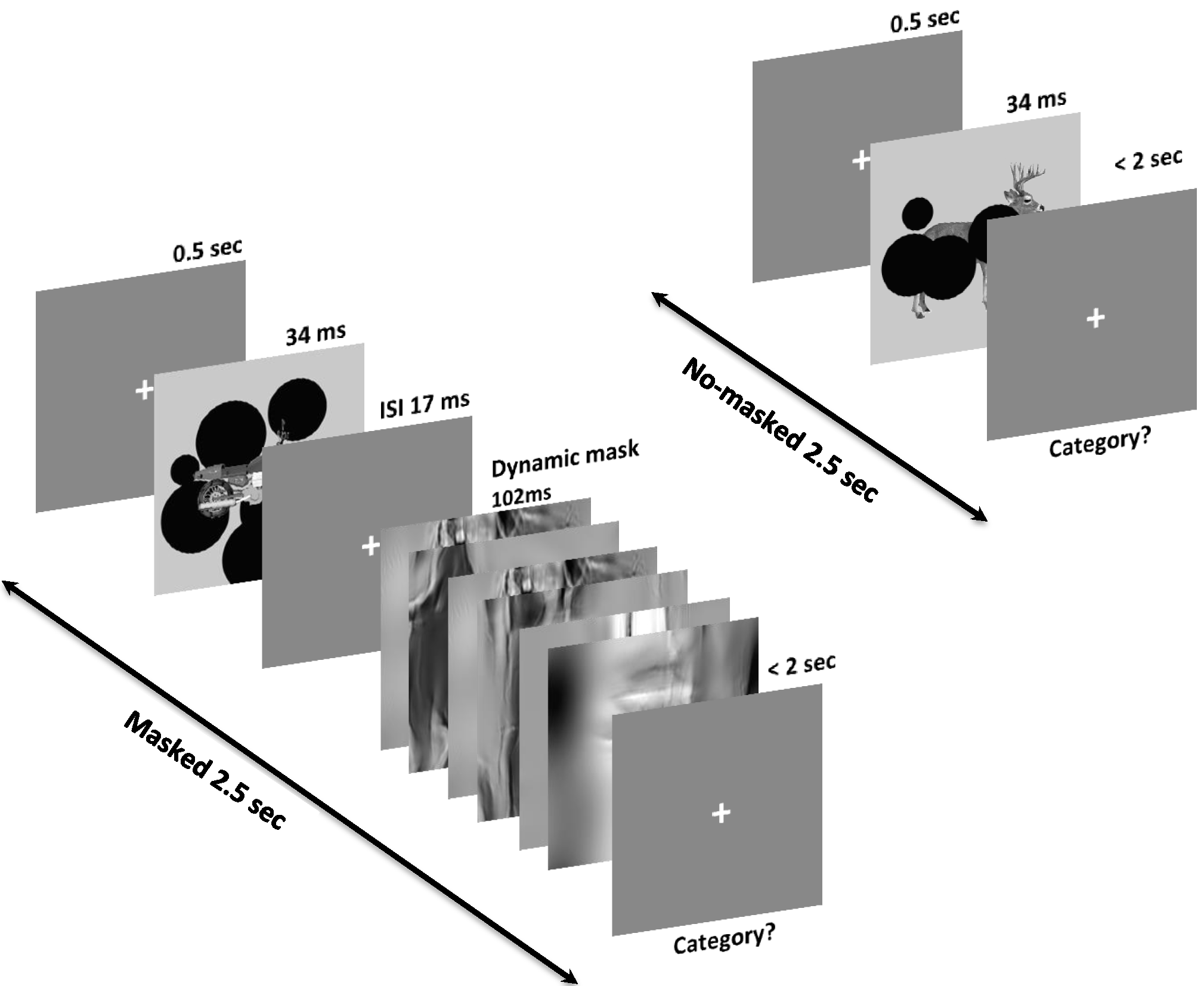

Supplement: S6 Fig — The behavioral experiment had two types of trials: mask and no mask trials (in random order). Each trial started by 0.5sec fixation, followed by a short presentation of stimulus for 34ms. In the masked trials, 17ms after the stimulus offset (short ISI) a dynamic mask of 100ms was presented. The dynamic mask was a sequence of synthesized images. The subjects were instructed to respond as soon and accurate as possible. Subject’s response was to categorize the presented image by pressing one of the four pre-specified keys on a keyboard corresponding to the four object categories (camel, deer, car, and motorcycle). (TIF) [file pcbi.1007001.s006.tif]

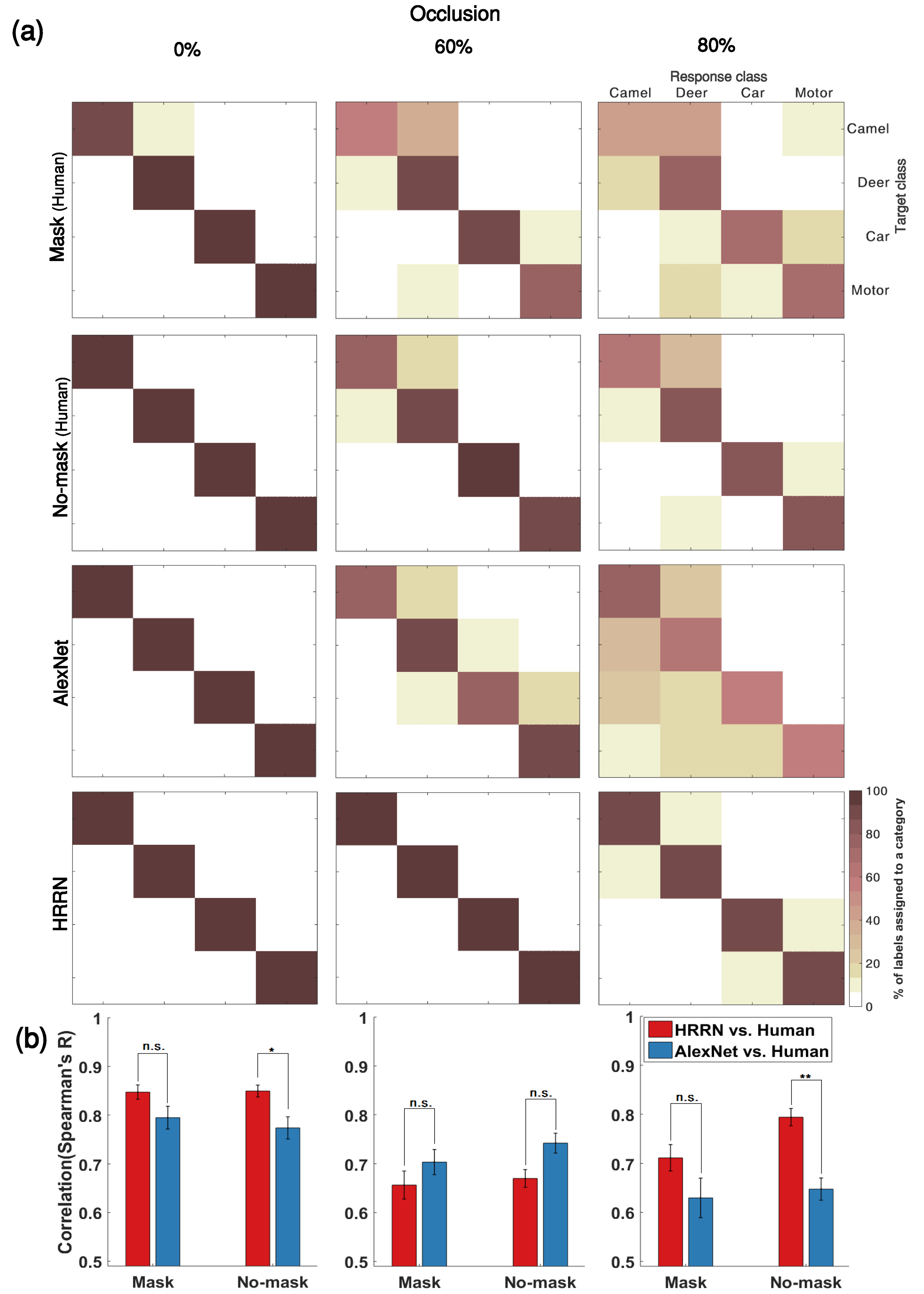

Supplement: S7 Fig — To compare patterns of errors in the models and humans, we computed confusion matrices. To obtain a confusion matrix, we first trained a SVM classifier on a multiclass object recognition task similar to the behavioral experiment. Then, we calculated the percentage of predicted labels assigned to a category. We display these percentages using color-codes in the matrix. Elements in the main diagonal of the confusion matrix show classification performances and off-diagonal elements show errors made in the classification. (a) Confusion matrices for the three levels of occlusion. The color bar, at the bottom-right corner, indicates the percentage of labels assigned to a category. (b) Bars indicate correlations between confusion matrices of the models with that of humans (mask and no-mask). Stars show significant differences between HRRN and AlexNet (signrank test, across subjects). * = p<0.05; ** = p<0.01. (TIF) [file pcbi.1007001.s007.tif]

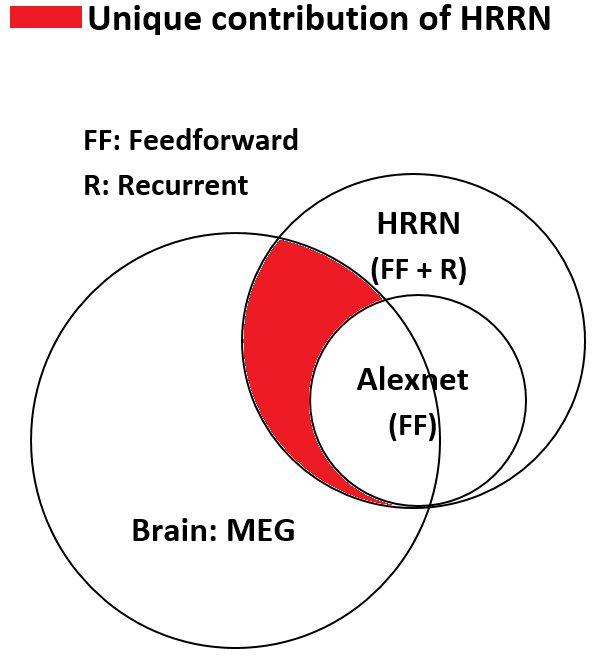

Supplement: S8 Fig — Red area indicates the unique contribution of HRRN in explaining MEG data. AlexNet has no unique contribution likely due to a component shared between the two models (i.e. feedforward component). (TIF) [file pcbi.1007001.s008.tif]

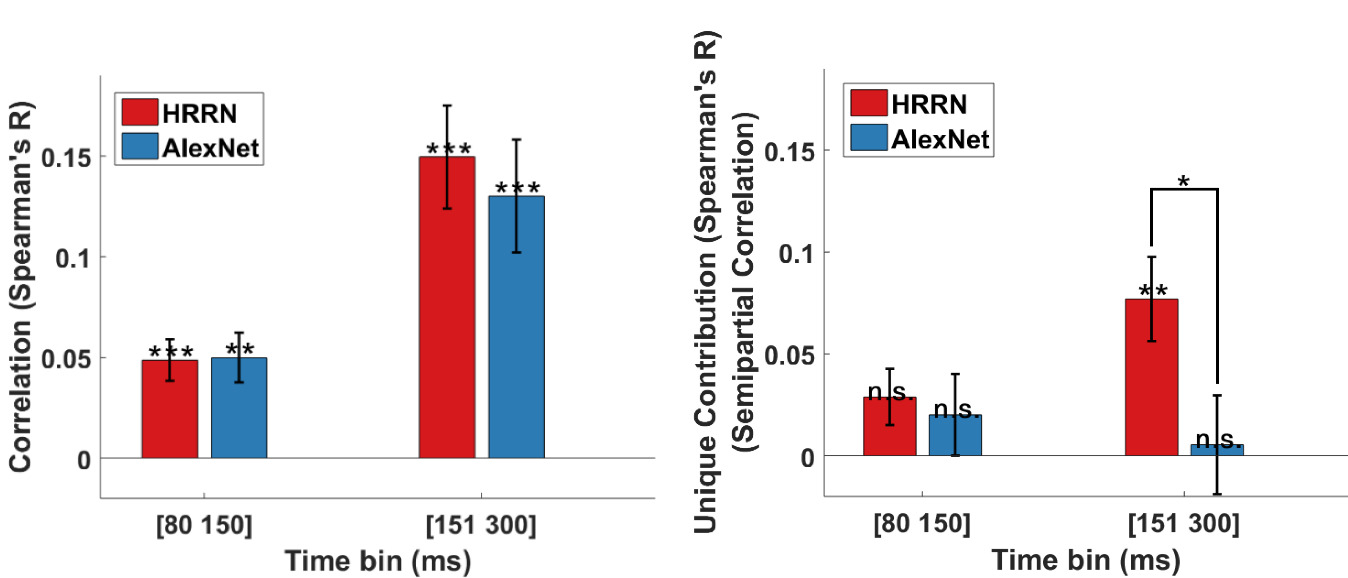

Supplement: S9 Fig — (a) Correlation between the models RDMs and the average MEG RDM over two different time bins. (b) Unique contribution of each model (semipartial correlation) in explaining the MEG data. Error bars represent SEM (Standard Error of the Mean). Significantly above zero correlations/semipartial-correlations and significant differences between the two models are indicated by stars. * = p<0.05; ** = p<0.01; *** = p<0.001. (TIF) [file pcbi.1007001.s009.tif]

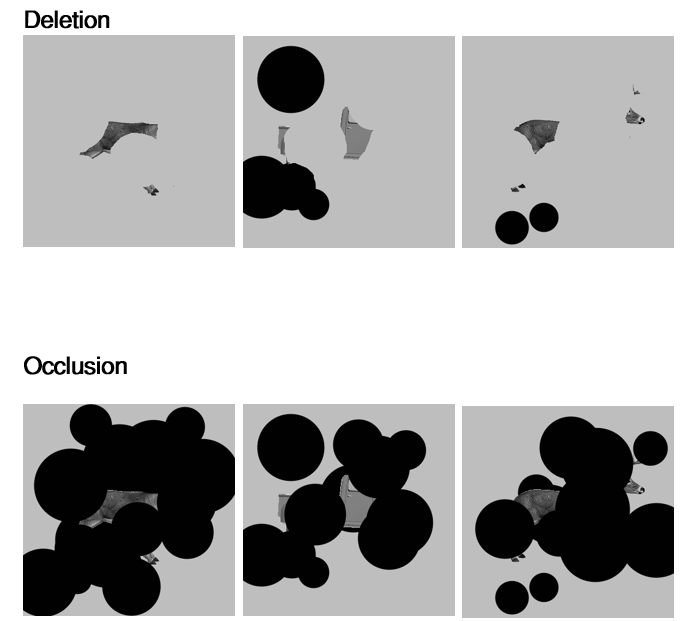

Supplement: S10 Fig — (TIF) [file pcbi.1007001.s010.tif]

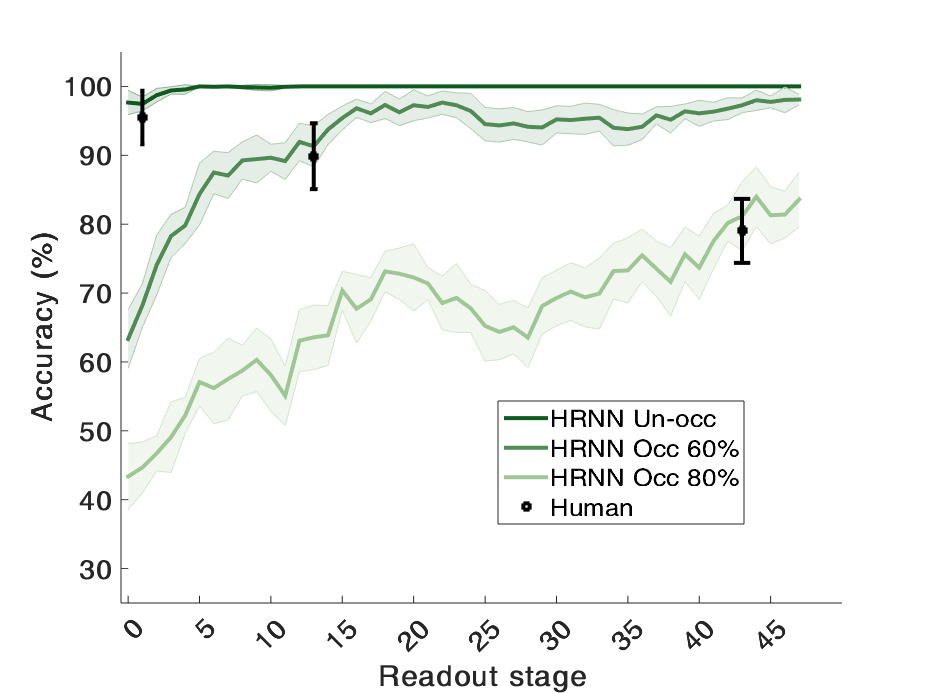

Supplement: S11 Fig — Shaded error bars indicate SD. Black circles are average accuracies across n = 16 human participants. Readout stage: readout stage refers to the number of local recurrent iterations involved in processing the input image throughout the hierarchy of the network. Readout stage 0 is when the model is fully feedforward (no local recurrent is active). And readout stage 1 is when only one recurrent iteration is engaged and readout stage n is when the network has gone through n recurrent iterations. (TIF) [file pcbi.1007001.s011.tif]

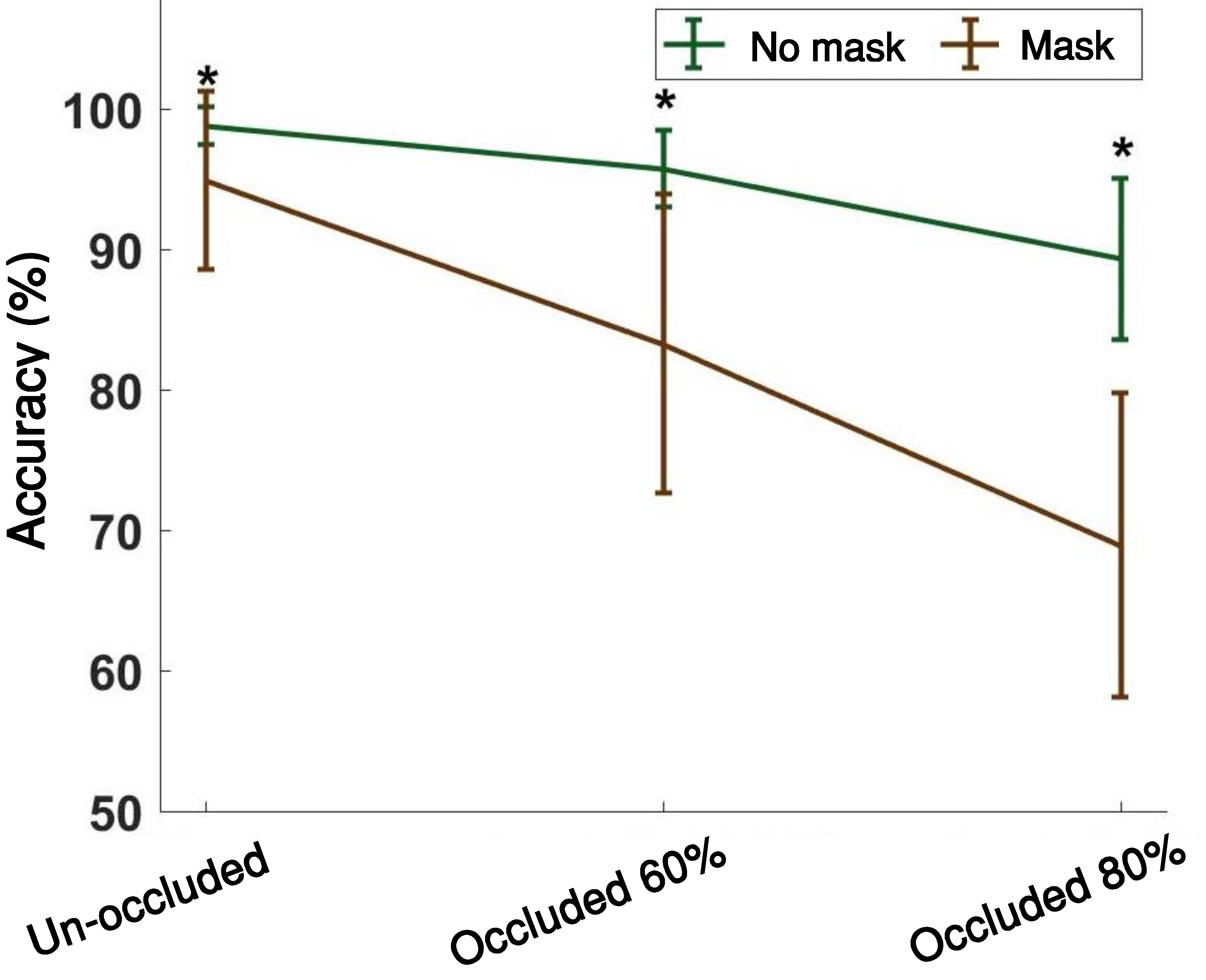

Supplement: S12 Fig — Stars indicate significant differences between mask and no-mask trials. The results are averaged over N = 15 human participants. (TIF) [file pcbi.1007001.s012.tif]

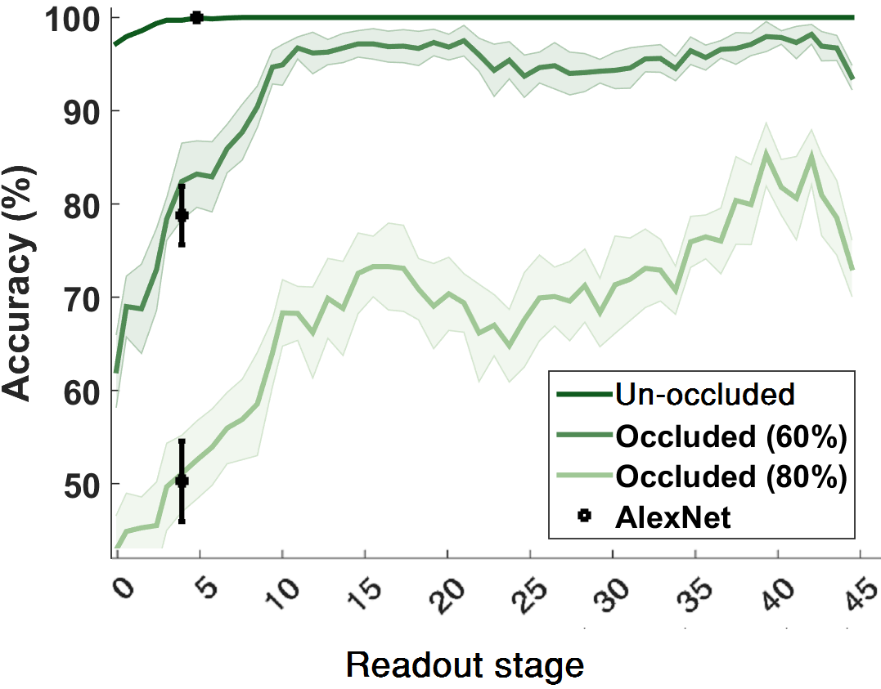

Supplement: S13 Fig — Shaded error bars indicate SD. Readout stage: readout stage refers to the number of local recurrent iterations involved in processing the input image throughout the hierarchy of the network. Readout stage 0 is when the model is fully feedforward (no local recurrent is active). And readout stage 1 is when only one recurrent iteration is engaged and readout stage n is when the network has gone through n recurrent iterations. Black circles are average accuracies for Alexnet, which are shown around the approximate corresponding HRRN readout stages. (TIF) [file pcbi.1007001.s013.tif]
